# Supplementary material for: Single‐cell sequencing maps gene expression to mutational phylogenies in PDGF‐ and EGF‐driven gliomas
Source: Mol Syst Biol. 2016 Nov 25;12(11):889. doi: 10.15252/msb.20166969 (PMC5147052; doi:10.15252/msb.20166969)
Supplement: Supplementary file 1 — Appendix [file MSB-12-889-s001.pdf]

Table of contents

1. Correlation of RNA-seq and exome-Seq
2. False discovery estimation of sc-CNV detection
3. Patient-specific SNVs
4. Versions of tools used in the analysis
5. Parameters used in the NGS processing pipelines

1. Correlation of RNA-seq and exome-seq

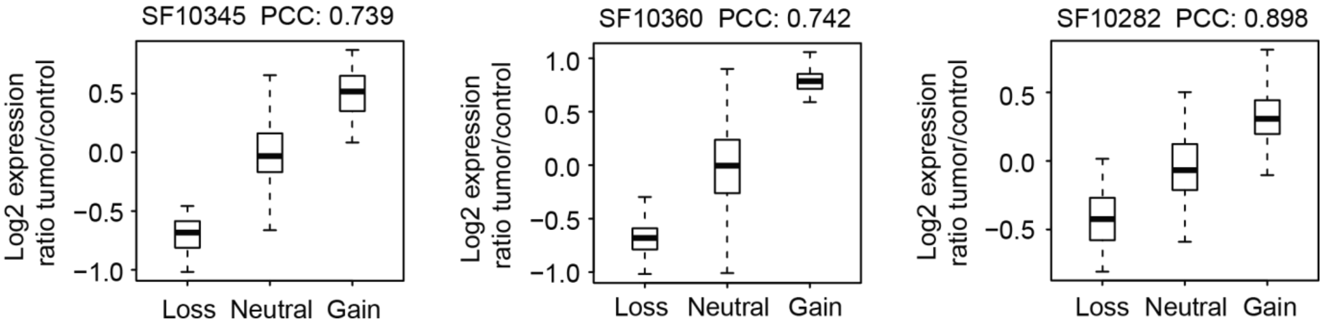

Figure S1: Distributions of GBM to normal-brain control single-cell expression ratios, across regions of copy number gain (exome-seq GBM/control ratio >1.3), neutral copy number, and loss (exome-seq GBM/control ratio<0.7). For each patient, read coverage of each gene was calculated for exome-seq and RNA-seq data. Genes covered by at least 100 reads by both methods were taken into account. Log2 ratios of GBM vs normal tissue across sliding windows comprising 200 genes was centered by the median (see Patel et al. 2014) and compared between RNA-seq and exome-seq. PCC stands for Pearson correlation coefficient. PCC was computed by comparing the vector of values obtained from the sliding window approach between single-cell RNA-seq and exome-seq.

2. False discovery rate estimation

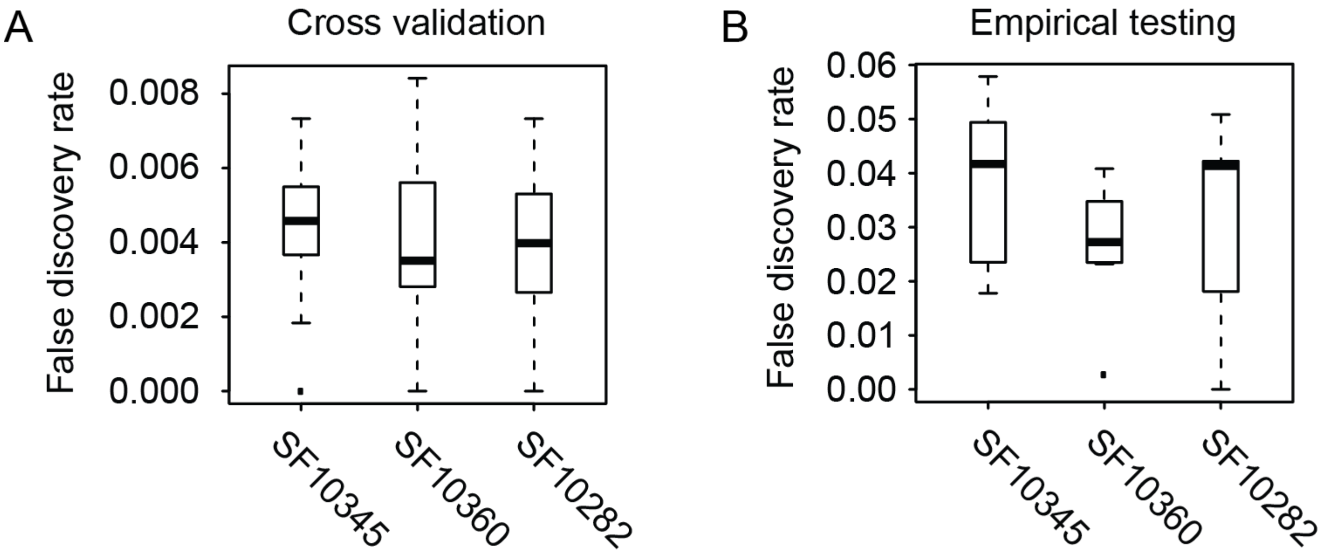

Figure S2: Distributions of the false discovery rates of CNV calls on single cell RNA-seq data. A) Cross validation on the dataset used as a normal brain control in the manuscript. We randomly selected 10%

of the cells as test, and the remaining 90% as training set. For each sample in the test set we generated CNV calls on each of the patient's altered regions defined by exome-seq. The false positive rate was calculated as: #positve CNV calls/#total CNV calls for each of the ten performed classification rounds. B) For each of the non-malignant cells from the dataset of Pollen et al. (2016) we generated CNV calls on each of the patient's altered regions defined by exome-seq. Error rates were calculated as described in A). For each set sample in Pollen et al., false positive rates were calculated independently.

3. Patient specific SNVs

Figure S3 Patient-specific SNVs. IGV screenshots for alignments of every patient's single cell RNA-seq (top), blood exome-seq (middle) and GBM exome-seq (bottom) at patient-specific germ-line mutations. Deviations from the reference (hg19) are indicated by color. VAF indicates the associated variant allele frequency.

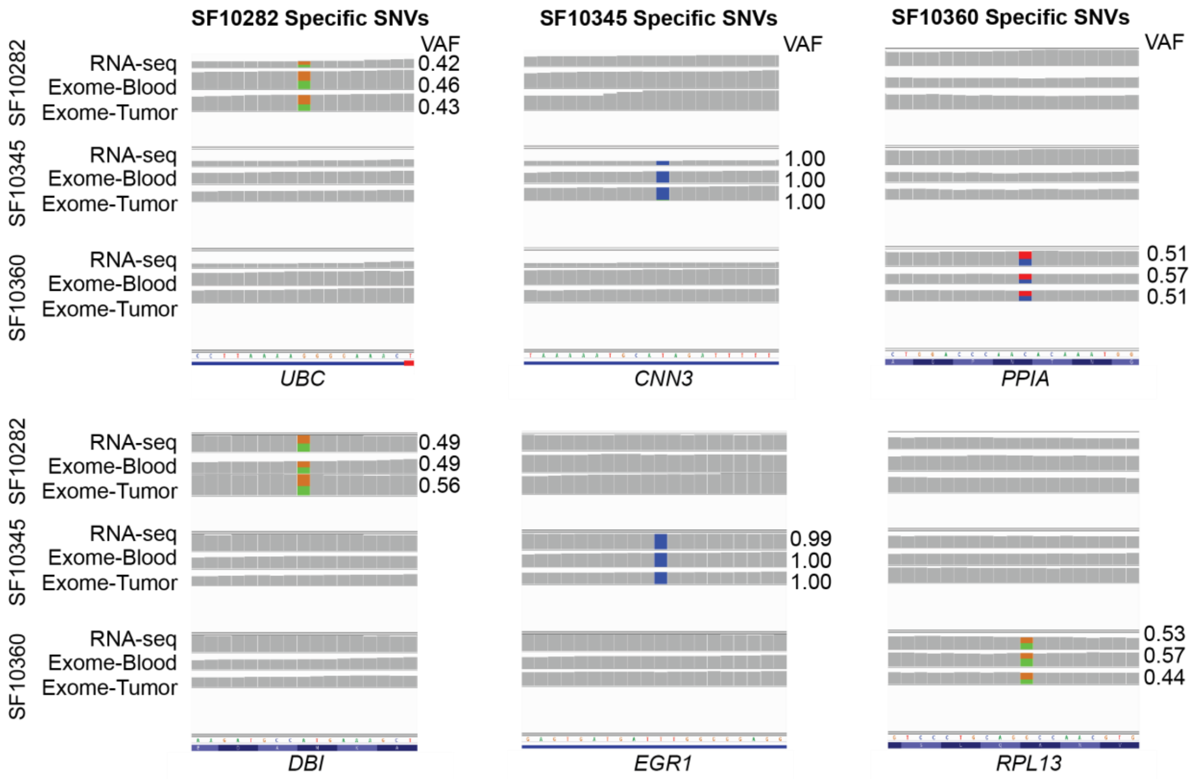

SF10282 Specific SNVs	SF10345 Specific SNVs	SF10360 Specific SNVs
3:69153854_ARL6IP5_G_T_heterozygous	15:40328292_SRP14_A_G_homozygous	19:582927_BSG_T_A_homozygous
7:5567112_ACTB_C_T_heterozygous	14:103986255_CKB_C_T_heterozygous	11:77790655_NDUFC2_G_C_homozygous
15:40328292_SRP14_A_G_heterozygous	14:90863489_CALM1_G_A_heterozygous	6:29913344_HLA-A_G_A_heterozygous
19:45978375_FOSB_A_G_homozygous	5:151042179_SPARC_A_C_heterozygous	14:102550803_HSP90AA1_G_A_homozygous
11:122928622_HSPA8_A_G_heterozygous	5:137804635_EGR1_T_C_homozygous	11:122928622_HSPA8_A_G_homozygous
21:35281393_ATP5O_A_G_heterozygous	8:26515663_DPYSL2_G_A_heterozygous	9:113006420_TXN_A_G_homozygous
17:7215536_EIF5A_T_C_heterozygous	16:56660841_MT1E_C_T_homozygous	17:32583911_CCL2_C_T_homozygous
3:69151152_ARL6IP5_C_A_heterozygous	1:156629037_BCAN_A_G_homozygous	11:122929407_HSPA8_T_G_heterozygous
21:35284683_ATP5O_A_G_heterozygous	16:56643343_MT2A_G_C_homozygous	6:29913374_HLA-A_C_G_heterozygous
1:44443854_ATP6V0B_G_A_heterozygous	16:56660816_MT1E_G_A_homozygous	7:44839429_PPIA_C_T_heterozygous
2:85133216_TMSB10_G_A_heterozygous	2:232573292_PTMA_T_C_homozygous	12:57106660_NACA_A_T_homozygous
2:120129841_DBI_A_G_heterozygous	1:95363001_CNN3_T_C_homozygous	17:79478007_ACTG1_G_A_homozygous
12:125396252_UBC_G_A_heterozygous	7:94059899_COL1A2_C_T_homozygous	6:44221404_HSP90AB1_G_C_heterozygous
6:44218120_HSP90AB1_A_G_homozygous	16:30081477_ALDOA_G_A_heterozygous	16:89628073_RPL13_G_A_heterozygous
12:76453966_NAP1L1_G_A_homozygous	8:27462481_CLU_A_G_heterozygous	17:32583269_CCL2_T_C_homozygous
2:29023749_PPP1CB_A_G_heterozygous	12:125397364_UBC_A_G_homozygous	
17:1303458_YWHAE_C_G_heterozygous	19:18685964_UBA52_G_T_heterozygous	
	17:7215536_EIF5A_T_C_homozygous	
	17:79478019_ACTG1_G_A_heterozygous	
	X:47444985_TIMP1_T_C_homozygous	
	2:232573301_PTMA_C_T_homozygous	

Table S1 Table of patient-specific SNVs giving their position (chr:pos), affected gene, reference and variant allele, as well as zygosity.

4. Versions of languages and tools used for the analysis

Annovar: Version: $Date: 2015-03-22 15:38:33 -0700 (Sun, 22 Mar 2015)
Bcftools: 1.2
BedTools: 2.17.0
BWA: 0.7.12
DESeq2: 1.10.1
FeatureCounts: 1.4.4
GATK Toolkit: 2.6-5
HISAT: 2.0.3
Java: 1.6.0_27
Mutect: 1.0.27783
Pindel: 0.2.4t
Python: 2.7
R: 3.2.1
SnpEff: 3.3
Tophat: 2.0.1
Trim-Galore: 0.3.8

5. Program calls of the NGS processing pipeline

A) Exome-Seq processing

I Data pre-processing and alignment

Initially, data was processed using the bcbio-nextgen pipeline (https://bcbio-nextgen.readthedocs.io/en/latest/index.html). The pipeline carries out all necessary steps including

alignment, filtering of low-coverage regions to increase sensitivity, re-alignment around indels, and re-calibration of quality scores. Attached are the two configuration files for the pipeline.

Sample.yaml


```yaml
fc_date: 20150824

fc_name: C7MMPANXX

upload:

  dir: SF10345

details:

  - files: [SF10345_R1.fastq, SF10345_R2.fastq]

    description: SF10345

    analysis: 'variant'

    genome_build: GRCh37

    lane: Sample_SF10345

    algorithm:

      aligner: bwa

      trim_reads: false

      recalibrate: true

      realign: true

      variantcaller: [gatk, gatk-haplotype]

      coverage_interval: exome

      coverage_depth: high

      hybrid_bait: SeqCap_EZ_Exome_v3_capture_GRC37.target.bed

      hybrid_target: SeqCap_EZ_Exome_v3_capture_GRC37.target.bed

      variant_regions: SeqCap_EZ_Exome_v3_capture_GRC37.target.bed

      clinical_reporting: true
```


system.yaml


```yaml
galaxy_config:/sequencing/src/galaxy-central/universe_wsgi.ini

program:

  bowtie: bowtie

  samtools: samtools

  tophat: tophat
```

```
  bwa: bwa
  ucsc_bigwig: wigToBigWig
  fastqc: fastqc
  pdflatex: pdflatex
  barcode: barcode_sort_trim.py
algorithm:
  aligner: bowtie
  max_errors: 2
  num_cores: 1
  platform: illumina
  recalibrate: false
  snpcall: false
  java_memory: 5g
  upload_fastq: false
  save_diskspace: false
  quality_format: Standard
  bc_position: 5
  bc_allow_indels: false
  bc_mismatch: 1
  bc_read: 1
  trim_reads: true
  #sv_detection: hydra
  num_gatk_threads: 8

analysis:
  towig_script: bam_to_wiggle.py
  process_program: automated_initial_analysis.py
  upload_program: upload_to_galaxy.py
  worker_program: nextgen_analysis_server.py
distributed:
```

```
  cluster_platform: lsf
  platform_args: '-q long_parallel -n 4 -R "rusage=[1500m]"'
  num_workers: 2
  rabbitmq_vhost: bionextgen
# Configuration algorithm changes for specific pipelines.
custom_algorithms:
  variant:
    aligner: bwa
    recalibrate: true
    recalibration_plots: false
    snpcall: true
    coverage_depth: "high" # other options: low
    coverage_interval: "exome" # other options: genome, regional
    train_hapmap: variation/hapmap_3.3.vcf
    train_1000g_omni: variation/1000G_omni2.5.vcf
    train_indels: variation/Mills_and_1000G_gold_standard.indels.vcf
    java_memory: 4g
    dbsnp: variation/dbsnp_137.vcf

resources:
  log:
    dir: /sequencing/cron_pipeline/std/SF10345
  ucsc_bigwig:
    memory: 36g
  bwa:
    cores: 1
    cmd: bwa
  novoalign:
    cores: 1
    memory: 4G
```

```
  gatk:
    jvm_opts: ["-Xms2g", "-Xmx4g", "-XX:+UseSerialGC"]
    dir: /sequencing/src/GenomeAnalysisTK-2.6-5-gba531bd/
  picard:
    jvm_opts: ["-Xms2g", "-Xmx4g"]
    dir: /sequencing/src/picard-1.93/dist/
  snpEff:
    jvm_opts: ["-Xms2g", "-Xmx6g"]
    dir: /sequencing/src/snpEff-3.3/
```

II Somatic SNV calling

Using the alignment file from I, we performed mutation calls as follows:

#Make raw mutect calls

```
java –Xmx8g -jar mutect.jar --analysis_type MuTect --logging_level WARN --
reference_sequence hg19.fa --intervals SeqCap_EZ_Exome_v3_hg19_primary_targets.bed
--input_file:normal SF10345_normal.bam --input_file:tumor SF10345_tumor.bam -baq
CALCULATE_AS_NECESSARY --out SF10345.snvs.raw.mutect.txt
```

#Estimate genotypes

```
 java –Xmx8g -jar GenomeAnalysisTK.jar --analysis_type UnifiedGenotyper --
genotype_likelihoods_model SNP --genotyping_mode DISCOVERY –input_file
SF10345_normal.bam --input_file SF10345_tumor.bam --reference_sequence hg19.fa --
dbsnp dbsnp_138.hg19.sorted.vcf -logging_level WARN --intervals SF10345.temp.bed  -
baq CALCULATE_AS_NECESSARY --noSLOD --standard_min_confidence_threshold_for_calling
30.0 --standard_min_confidence_threshold_for_emitting 10.0 --min_base_quality_score
20 --output_mode EMIT_VARIANTS_ONLY --out SF10345.UG.snps.raw.vcf
```

#Annotate variants

```
java -Xmx8g -jar GenomeAnalysisTK.jar --analysis_type VariantAnnotator  --
input_file SF10345_normal.bam --input_file SF10345_tumor.bam --reference_sequence
hg19.fa --dbsnp dbsnp_138.hg19.sorted.vcf --logging_level WARN  --intervals
SF10345.UG.snps.raw.vcf  --variant SF10345.UG.snps.raw.vcf  -baq
CALCULATE_AS_NECESSARY  --annotation QualByDepth  --annotation RMSMappingQuality --
annotation MappingQualityZero --annotation LowMQ --annotation
MappingQualityRankSumTest --annotation FisherStrand  --annotation HaplotypeScore –
annotation ReadPosRankSumTest --annotation DepthOfCoverage –out
SF10345.UG.snps.annotated.vcf
```

#Filter annotated variants

```
java -Xmx8g -jar GenomeAnalysisTK.jar --analysis_type VariantFiltration --
reference_sequence hg19.fa --logging_level WARN --variant
SF10345.UG.snps.annotated.vcf -baq CALCULATE_AS_NECESSARY --filterExpression
"QD < 2.0" --filterName QDFilter --filterExpression "MQ < 40.0" --filterName
MQFilter --filterExpression "FS > 60.0" --filterName FSFilter --
filterExpression "HaplotypeScore > 13.0" --filterName HaplotypeScoreFilter -
-filterExpression "MQRankSum < -12.5" --filterName MQRankSumFilter --
filterExpression "ReadPosRankSum < -8.0" --filterName ReadPosFilter --out
SF10345.UG.snps.filtered.vcf
```

III Somatic small Indel calling

Call somatic indels from BAM alignments of control and tumor exome-seq reads

#Make raw pindel calls

```
pindel -f hg19.fa -i SF10345_pindel.cfg -c ALL -o SF10345.pindel -r FALSE -t FALSE
-l FALSE -k FALSE -T 12
```

#Parse to vcf

```
pindel2vcf -P SF10345.pindel -r hg19.fa -R hg19 -d 20121031 -v SF10345.pindel.vcf -
G
```

#Filter raw calls based on coverage, alignment quality and supporting reads

```
python pindel_filter.py SF10345.pindel.vcf SF10345.pindel.filter
```

#Only keep variants overlapping genes

```
bedtools intersect -a SF10345.pindel.filter -b
SeqCap_EZ_Exome_v3_hg19_primary_targets.bed -wa > SF10345.filter.intersect
```

#Filter against DBSnP

```
perl annotate_variation.pl -filter -dbtype snp138 -buildver hg19
SF10345.filter.intersect humandb/
```

#Annotate variants

```
perl annotate_variation.pl --geneanno --buildver hg19 --outfile
SF10345.filter.intersect.anno SF10345.filter.intersect
/diazlab/shared/annovar/humandb/
```

IV Somatic CNV calling

#Run ADTEX pipeline

```
python ADTEx.py --normal SF10345_normal.bam --tumor SF10345_tumor.bam --bed
SeqCap_EZ_Exome_v3_hg19_primary_targets.bed --out SF10345_CNVs.txt
```

B) Single cell RNA-Seq processing

I Read quantification

#Removed adapters and low quality bases

```
trim_galore -q 20 --nextera --length 20 -o ./tmp --paired C1_r1.fq C1_r2.fq
```

#Map reads to human genome with tophat2

```
tophat2 -o C1.bam -p 24 --transcriptome-index=transcriptome/refSeq --prefilter-
multihits genome/hg19 C1_r1.fq C1_r2.fq
```

#Count uniquely mapped reads

```
featureCounts -T 18 -p -C -a genes_ercc.gtf -o SF10345_counts.txt *.bam
```

II SNV calling (Experimental code, output not used in manuscript)

#Determine origin for sets of reads in order to call genotypes

```
java -Xmx2g -jar picard.jar AddOrReplaceReadGroups I=C1.bam O=C1.flt.bam
SO=coordinate RGID=1 RGLB=1 RGPL=illumina RGPU=1 RGSM=1
VALIDATION_STRINGENCY=SILENT
```

#Remove PCR duplicates

```
java -Xmx2g -jar picard.jar MarkDuplicates I=C1.flt.bam O=C1.flt.rmdup.bam
CREATE_INDEX=true VALIDATION_STRINGENCY=SILENT M=C1.metrics
rm -f C1.flt.bam
```

#Order by genomic pos

```
java -Xmx2g -jar picard.jar ReorderSam I=C1.flt.rmdup.bam
O=C1.flt.rmdup.reorder.bam  R=hg19.fa VALIDATION_STRINGENCY=SILENT
rm -f 1.flt.rmdup.bam
```

#Index sorted BAM

```
samtools index C1.flt.rmdup.reorder.bam
```

#Run GATK to creat two reads from one spliced read

```
java -Xmx2g -jar GenomeAnalysisTK.jar -T SplitNCigarReads -R hg19.fa -I
C1.flt.rmdup.reorder.bam -o C1.split.bam -fixNDN -rf ReassignOneMappingQuality -
RMQF 255 -RMQT 60 -U ALLOW_N_CIGAR_READS
rm -f C1.flt.rmdup.reorder.bam
```

#Index BAM file

```
samtools index C1.split.bam
```

#Run GATK toolkit for SNV detection

```
java -Xmx2g -jar GenomeAnalysisTK.jar -T HaplotypeCaller -R hg19.fa -I C1.split.bam
-dontUseSoftClippedBases -stand_call_conf 20.0 -stand_emit_conf 20.0 -o C1.vcf
rm -f C1.split.bam
```

#Annotate variants

```
perl table_annovar.pl C1.vcf humandb/ -buildver hg19 -out C1_anno.vcf -remove -
tempdir /scratch -protocol refGene,snp138NonFlagged,ljb26_all,cosmic70,gerp++gt2 -
operation g,f,f,f,f -nastring . -vcfinput
```

#Filter for only exonic SNVs not in DBSNP, having a strong SIFT score

```
bcftools view C1_anno.vcf | egrep
"Func.refGene=exonic|Func.refGene=UTR|cosmic70=."|grep PASS|grep
'snp138NonFlagged=\.'|egrep 'SIFT_pred=D|SIFT_pred=P'>tmp1
```

```
bcftools view -h C1_anno.vcf >tmp.hdr
cat tmp.hdr tmp1>tmp2
bcftools view -o C1_anno.pmut.bcf -O b tmp2
bcftools index C1_anno.pmut.bcf
rm -f tmp*
```

C) Bulk population RNA-Seq processing

I Read quantification

#Map reads to human genome with hisat2

```
hisat2 -p 2 --rna-strandness R –x grch37_snp_tran/genome_snp_tran -U smp1.fastq.gz
| samtools view -bSu - | samtools sort – smp1
```

#Count uniquely mapped reads

```
featureCounts -T 18 –s 2 –t exon –g gene_id -a genes.gtf -o counts.txt *.bam
```
