# Supplementary material for: Single‐cell sequencing maps gene expression to mutational phylogenies in PDGF‐ and EGF‐driven gliomas
Source: Mol Syst Biol. 2016 Nov 25;12(11):889. doi: 10.15252/msb.20166969 (PMC5147052; doi:10.15252/msb.20166969)
Supplement: Supplementary file 2 — Expanded View Figures PDF [file MSB-12-889-s002.pdf]

Expanded View Figures

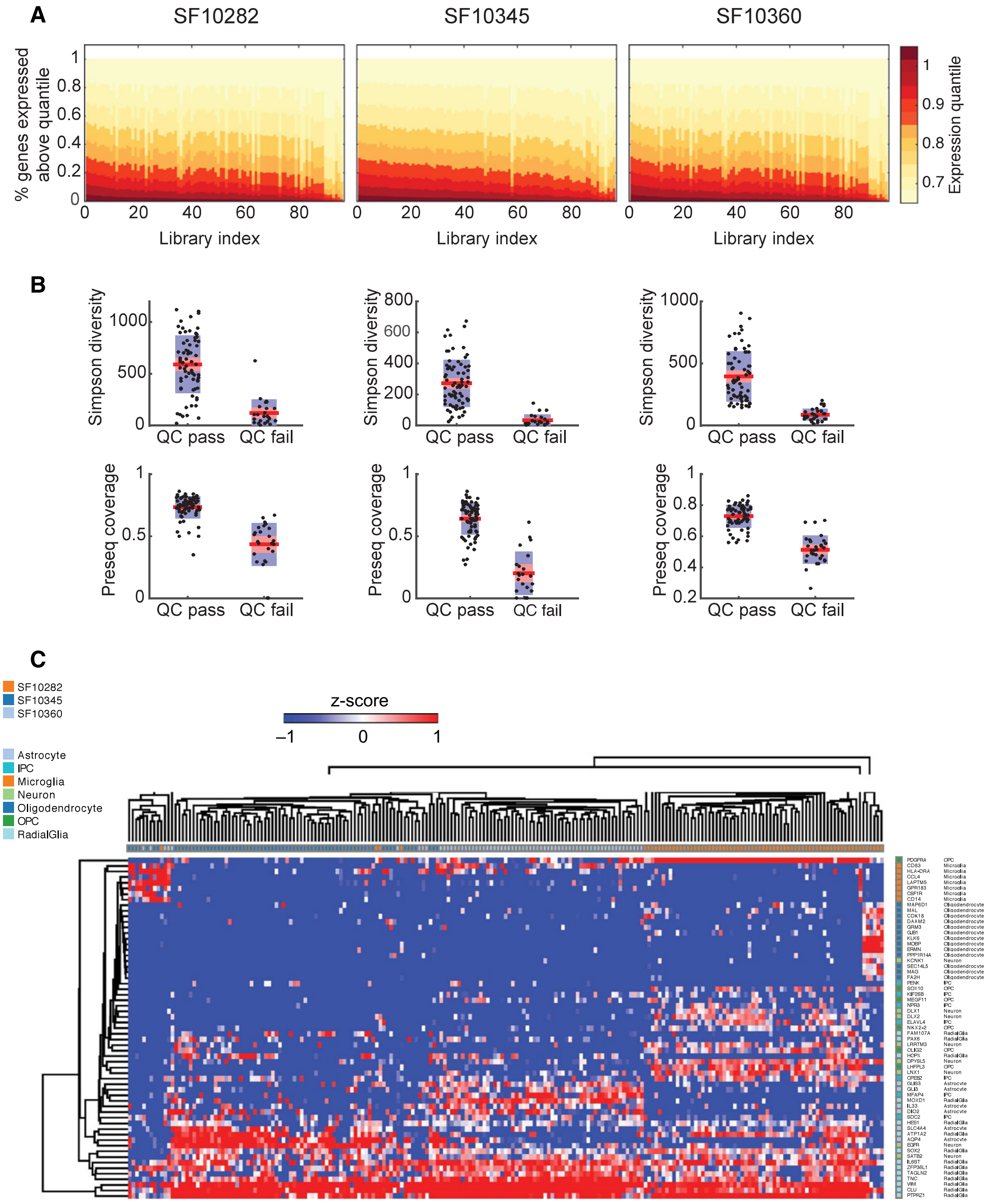

Figure EV1. Quality control and preprocessing.

A Percent of genes expressed above a given expression percentile for each case.

B Simpson diversity scores and Preseq coverage for each sample.

C Hierarchical clustering of all cells, using a biomarker panel of genes expressed by cell types found in the brain.

 © 2016 The Authors

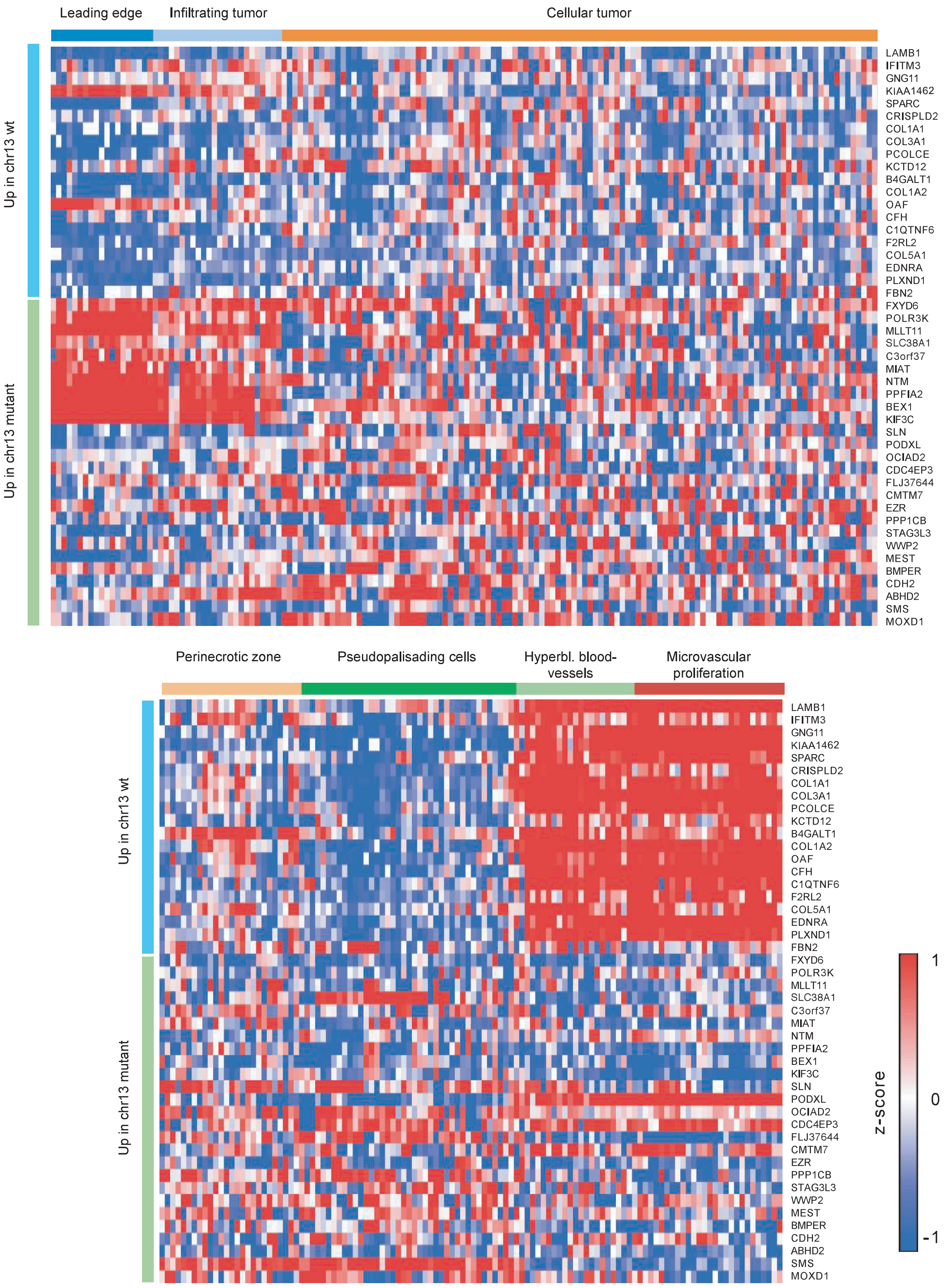

Figure EV2. Expression of genes differentially expressed between the chromosome 13 loss clone, and the wild-type clone in SF10360, in the Ivy Glioblastoma Atlas.

Gene expression across all samples in the Ivy Glioblastoma Atlas, for genes with and adjusted *P*-value < 0.05 in the differential analysis between cells harboring the chr13 loss and those free of CNV alterations on chr13.

© 2016 The Authors

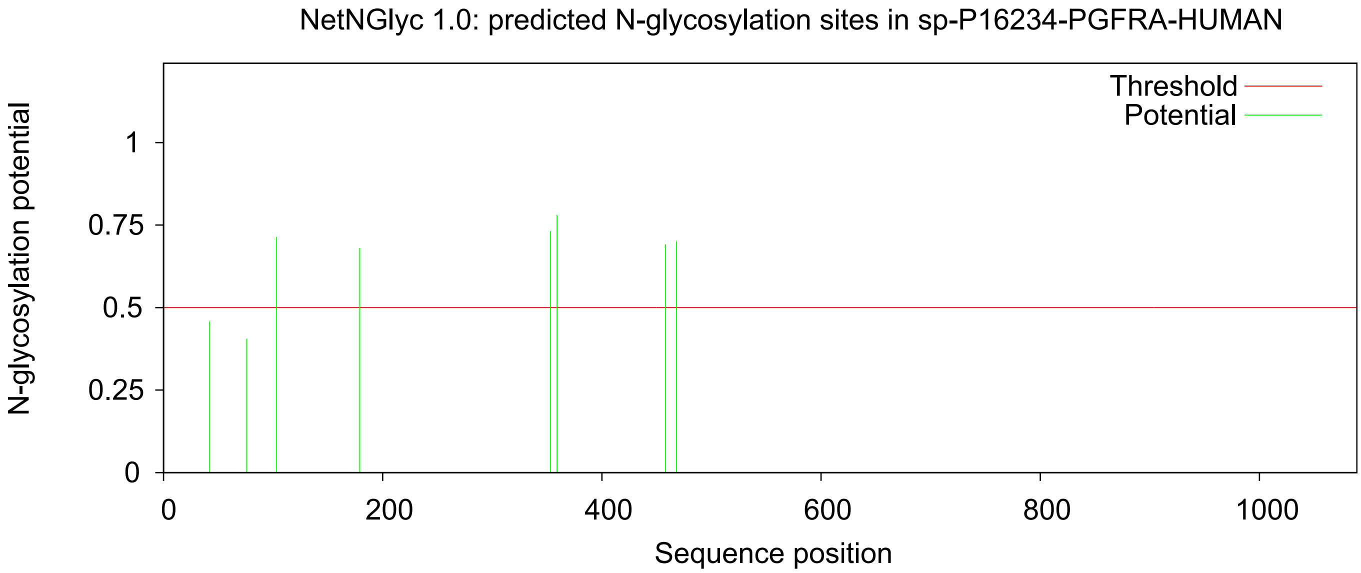

Figure EV3. Predicted glycosylation sites in PDGFRA.
Glycosylation sites in PDGFRA, predicted by NetNGly 1.0.

 © 2016 The Authors
